# Effects of process parameters on sheet resistance uniformity of fluorine-doped tin oxide thin films

**DOI:** 10.1186/1556-276X-7-17

**Published:** 2012-01-05

**Authors:** Chairul Hudaya, Ji Hun Park, Joong Kee Lee

**Affiliations:** 1 Advanced Energy Materials Processing Laboratory, Energy Storage Research Center, Korea Institute of Science and Technology, Hwarangno 14 gil 5, Seongbuk-gu, Seoul, 136-791, Republic of Korea; 2 Department of Clean Energy and Chemical Engineering, University of Science and Technology, 176 Gajungro Yuseong-gu, Daejeon, 305-350, Republic of Korea; 3 Department of Advanced Material Engineering, Korea University, Seongbuk-gu, Seoul, 136-701, Republic of Korea

**Keywords:** process parameters, FTO, thin film, ECR-MOCVD, sheet resistance uniformity

## Abstract

An alternative indium-free material for transparent conducting oxides of fluorine-doped tin oxide [FTO] thin films deposited on polyethylene terephthalate [PET] was prepared by electron cyclotron resonance - metal organic chemical vapor deposition [ECR-MOCVD]. One of the essential issues regarding metal oxide film deposition is the sheet resistance uniformity of the film. Variations in process parameters, in this case, working and bubbler pressures of ECR-MOCVD, can lead to a change in resistance uniformity. Both the optical transmittance and electrical resistance uniformity of FTO film-coated PET were investigated. The result shows that sheet resistance uniformity and the transmittance of the film are affected significantly by the changes in bubbler pressure but are less influenced by the working pressure of the ECR-MOCVD system.

## Introduction

Transparent conducting oxide [TCO] has been used in many applications, such as solar cells, flat panel displays, and smart windows [1]. Indium tin oxide [ITO] is used most widely for TCOs, owing to its high electrical conductivity and transparency properties. On the other hand, the indium price has soared recently due to the rapid demand of ITO [2]. To replace indium, many materials have been proposed for TCO of which one of them is fluorine-doped tin oxide [FTO].

Sheet resistance uniformity of thin films deposited on TCO substrates is one of the key successful parameters of the film quality. Previous studies examined ways of achieving a high uniformity of deposit using a range of different deposition techniques, such as pulse laser deposition [3], chemical beam coating [4], vacuum arc deposition [5], and plasma enhanced chemical vapor deposition. In addition, some studies also simulated the uniformity models of the deposit through a Monte Carlo simulation [6,7]. All of them used glass as substrate, but it has been suggested that the deposition of thin films on plastic substrates, such as polyethylene terephthalate [PET], is still lacking due to limitations of the polymer physical properties, which requires a low-temperature deposition [8].

Compared to glass, PET has its advantages because it is flexible and elastic, which can make PET be shaped for many applications. One of the deposition methods that can allow low-temperature deposition is electron cyclotron resonance - metal organic chemical vapor deposition [ECR-MOCVD]. ECR-MOCVD can deposit a thin film at room temperature, which will not deform a plastic substrate.

Depending on the techniques for growing the thin film, the process parameters such as working and bubbler pressures significantly influence the quality of the thin film [1,3,9]. This study deeply examines the effects of those parameters in ECR-MOCVD on sheet resistance uniformity and transmittance of an FTO thin film coated on a PET substrate.

## Experimental method

**Figure 1:**
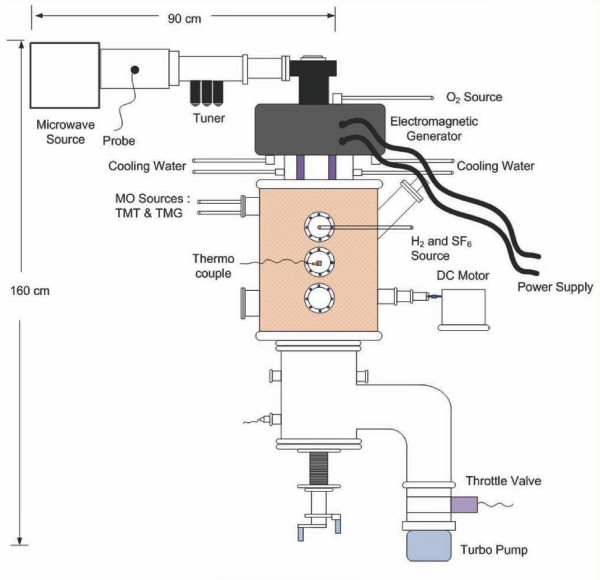
**A schematic of ECR-MOCVD**. ECR-MOCVD consists of an electromagnetic generator, microwave power source, and plasma chamber, which is supported by vacuum-pump equipment.

The ECR system consists mainly of two separate regions, i.e., the plasma source and the deposition zone [10]. Cyclotron resonance is achieved when the frequency of the alternating electric field is made to match the natural frequency of the electrons orbiting the lines of force. This phenomenon occurs when the microwave source with a frequency and maximum power of 2.45 GHz and 1,400 W, respectively, is introduced to the plasma chamber through a rectangular waveguide under a static magnetic flux density of 875 G. A turbo molecular pump was used to produce a vacuum. A 16 × 34 cm^2 ^PET substrate was treated under a range of experimental conditions, as shown in Table 1. The plasma-assisted deposition lasted for about 15 min. Tetra methyl tin [TMT] and sulfur hexafluoride [SF_6_] were used as the tin and fluorine precursors, respectively. The flow rates of gasses were controlled by mass flow rate controllers, and TMT and SF_6 _were introduced to the chamber by argon gas as the carrier. Figure 1 shows the schematic diagram of ECR-MOCVD.

**Table 1:** Details of the experimental conditions

Variable parameters	Unit	Value	Fixed parameters	Unit	Value
Working pressure	mTorr	5	Sample area	cm**^2^**	16 × 34
		7.5			
		10			
		12.5			
		15			
			H**_2 _**flow rate	sccm	5.2
Bubbler pressure	Torr	40.2	O**_2 _**flow rate	sccm	36.4
		43.3			
		45.7			
		60.6			
			TMT flow rate	sccm	3.6
			SF**_6 _**flow rate	sccm	12.46
			Ar flow rate	sccm	12.18
			Deposition time	min	15

A four-point probe was used to measure the sheet resistance at 16 points that are fairly distributed on the samples. To observe the optical properties, a UV visible spectrophotometer was used to quantify the transmittance of the FTO thin film deposited on PET.

## Results and discussion

The process parameters examined in this study are the working and bubbler pressure variations. The working pressure is defined as the pressure applied in the plasma chamber of ECR-MOCVD, whereas the bubbler pressure is described as the pressure introduced to the TMT. Each of these parameters was examined by five different experiments with the conditions as listed in Table 1.

### Sheet resistance and transmittance of FTO film

A higher transmittance and a lower sheet resistance of the thin film are desirable for TCO material applications. On the other hand, there is a trade-off among the two parameters. Generally, a lower sheet resistance will be obtained when the thin film is thick enough, which causes degeneracy on the transmittance.

Both parameters are correlated as the figure of merit [9], which represents the TCO performance as follows:

(1) $$\emptyset_{\text{TC}}=\frac{T}{\text{Rs}}$$

where *T *is the total visible transmittance at *λ *= 550 nm (%) and Rs is the sheet resistance (Ω/sq).

In this paper, the samples with a size of 16 × 34 cm^2 ^were examined by a four-point probe for each set of *x *and *y *coordinates, i.e., (1, 1), (4, 1), (8, 1), (12, 1), (1, 10), (4, 10), (8, 10), (12, 10), (1, 20), (4, 20), (8, 20), (12, 20), (1, 30), (4, 30), (8, 30), and (12, 30). The sheet resistance uniformity is defined based on the equation:

(2) $$\text{UNI }\left(\text{\% }\right)=\frac{\left(\text{R}{\text{s}}_{\text{Max}}-\text{R}{\text{s}}_{\text{Min}}\right)}{2\times \text{R}{\text{s}}_{\text{Average}}}\;\times 100\text{\% }$$

(3) $$\text{Rs}=\frac{\rho }{t}$$

where UNI (%) is the sheet resistance uniformity; Rs_Max_, Rs_Min_, and Rs_Average_, *ρ*, and *t *are highest, lowest, and average of measured sheet resistance (ohm/square), resistivity (ohm.cm), and film thickness (cm), respectively. Equation 2 indicated that the smaller UNI (%) is preferable. Meanwhile, the higher UNI (%) represents a higher ranging value between Rs_Max _and Rs_Min _which means that the film has a poor sheet resistance uniformity.

Because other process parameters of the deposition are fixed, the film thickness growth under our ECR-MOCVD system is solely controlled by the bubbler pressure, as explained in a previous work [1]. The prepared film thickness is ranging from 300 to 450 nm in our experimental range.

### Effects of bubbler pressure variation

**Figure 2:**
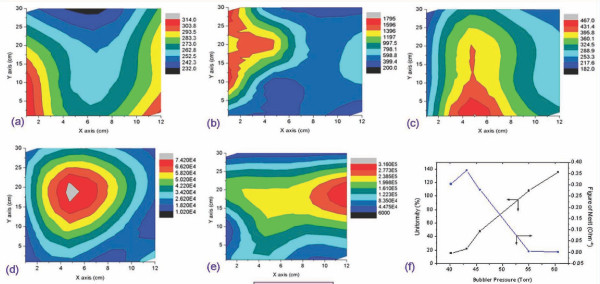
**Contour maps of an FTO thin film on a PET substrate for bubbler pressure variations**. The bubbler pressures were varied at (**a**) 40.2 Torr, (**b**) 43.2 Torr, (**c**) 45.7 Torr, (**d**) 55 Torr, (**e**) 60.2 Torr. (**f**) The sheet resistance uniformity and figure of merit of the FTO thin film under bubbler pressure variations.

Figures 2a to 2e show the contour of the sheet resistance uniformity of each bubbler pressure variation of 40.2, 43.3, 45.7, 55, and 60.6 Torr, respectively. The contour maps illustrate the regions where the sheet resistances of each bubbler pressure variation are mapped. The maps show the uniformity patterns that are formed in parallel with the *y*-axis obviously due to the application of a rolling system as a substrate holder in the ECR-MOCVD apparatus. The sheet resistance uniformity is likely to be present at the center of the coated PET substrate indicated by the larger areas with the same color. These phenomena were also confirmed by previous researchers [11].

The evolution of fluorine [F^-^] atoms to tin oxide films has contributed to the substitution of oxygen O^2- ^anions in the lattice and produces more free electrons, resulting in a decrease in sheet resistance [9]. On the other hand, in the present study, the gas flow rate of the fluorine precursor (SF_6_) and oxygen entering the plasma chamber were fixed at 12.46 sccm and 36.4 sccm, respectively. Therefore, there were no influences from the fluorine concentration to the sheet resistances of the thin film. As shown in Figure 2f, under bubbler pressure variations, the sheet resistance uniformity tends to increase with increasing bubbler pressure. The sheet resistance was dramatically increased when the pressure introduced was > 45.7 Torr. The rapid increase in sheet resistance can be explained by the chemical kinetics, where a higher bubbler pressure results in a higher flow rate of the TMT/Ar gas and increases the concentration of tin atoms in the plasma chamber. The maximum sheet resistance was 315,600 Ω/sq when the bubble pressure was 60 Torr after 15 min of deposition. Besides increasing the sheet resistance of the film, the increasing bubbler pressure also penetrates the nonuniformity of deposition. This can be observed as a large parity between the maximum and minimum sheet resistances.

**Figure 3:**
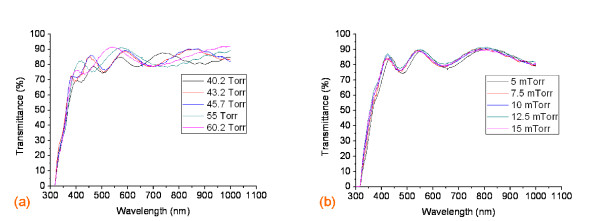
**The optical transmittance of an FTO thin film coated on a PET substrate**. Optical transmittance for (**a**) bubbler pressure variations and (**b**) working pressure variations.

In terms of the optical properties, we found that the increasing bubbler pressure affected the slightly higher film transmittance as shown in Figure 3a. At a wavelength of 550 nm, the transmittances of 40.2 and 60 Torr were 82.85% and 91.26%, respectively while the other transmittances of bubbler pressure variations were in between. This situation follows the figure of merits as described in Equation 1; hence, one has to compromise the two parameters in order to obtain the appropriate TCO materials.

### Influences of working pressure variation

**Figure 4:**
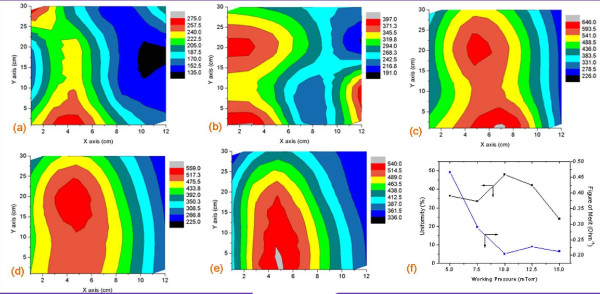
**Contour maps of an FTO thin film on a PET substrate for working pressure variations**. The working pressures were varied at (**a**) 5 mTorr, (**b**) 7.5 mTorr, (**c**) 10 mTorr, (**d**) 12.5 mTorr, and (**e**) 15 mTorr. (**f**) The sheet resistance uniformity and figure of merit of the FTO thin film under working pressure variations.

Figure 4a to 4e show the contour maps for the experiments examining the working pressure variations ranging from 5 to 15 mTorr. The maps suggest that the changes in working pressure did not affect the sheet resistance of the thin film significantly. The working pressure of 10 mTorr contributed to the highest average sheet resistance and was identified as having a larger sheet resistance uniformity area in the center of the sample. In the same manner as the sheet resistance, the transmittance of the FTO film coated on PET was not really dependent on the changes in the working pressure of the apparatus; in this case, the transmittance was similar (approximately 90%) as shown in Figure 3b.

## Conclusion

The effects of process parameter variations, in this case, the bubbler and working pressures, on sheet resistance uniformity and optical transmittance of FTO deposition on a PET substrate prepared by ECR-MOCVD were investigated. We found that the sheet resistance uniformity and transmittance of the film were significantly affected by the changes in bubbler pressure but were less influenced by the working pressure of the ECR-MOCVD system. The sheet resistance uniformity tends to increase with increasing bubbler pressure. The measurements carried out using a four-point probe showed that bubbler pressures > 45.7 Torr cause a dramatic increase in the sheet resistance of the film. The maximum sheet resistance was 315,600 Ω/sq at a bubbler pressure of 60 Torr after 15 min of deposition. At this point, the transmittance was excellent, i.e., 91.26%. This could be understood as the figure of merit of the TCO materials where both the sheet resistance and transmittance of the films can be compromised. For working pressure variations, a pressure of 10 mTorr contributed to the highest average sheet resistance and had a larger sheet resistance uniformity area in the center of the sample. Overall, suitable process parameters are needed to obtain the desired sheet resistance and transmittance of FTO films deposited on a PET substrate.

## Competing interests

The authors declare that they have no competing interests.

## Authors' contributions

All the authors contributed to the writing of the manuscript. JHP carried out the experiments under the command of JKL. All authors read and approved the final manuscript.
